# Marine Bacterial Community Structures of Selected Coastal Seawater and Sediment Sites in Qatar

**DOI:** 10.3390/microorganisms11122827

**Published:** 2023-11-21

**Authors:** Shimaa S. El-Malah, Kashif Rasool, Khadeeja Abdul Jabbar, Muhammad Umar Sohail, Husam Musa Baalousha, Khaled A. Mahmoud

**Affiliations:** 1 Qatar Environment and Energy Research Institute (QEERI), Hamad Bin Khalifa University, Qatar Foundation, Doha P.O. Box 34110, Qatar; salielmallah@hbku.edu.qa (S.S.E.-M.); krasool@hbku.edu.qa (K.R.); kabduljabbar@hbku.edu.qa (K.A.J.); 2 Proteomics Core, Weill Cornell Medicine, Doha P.O. Box 24144, Qatar; mus4008@qatar-med.cornell.edu; 3 Department of Geosciences, College of Petroleum Engineering and Geosciences, King Fahd University of Petroleum and Minerals (KFUPM), Dhahran 31261, Saudi Arabia; husam.baalousha@kfupm.edu.sa

**Keywords:** marine, bacteria, diversity, seawater, sediment, arabian gulf

## Abstract

Severe environmental conditions can have a diverse impact on marine microorganisms, including bacteria. This can have an inevitable impact on the biofouling of membrane-based desalination plants. In this work, we have utilized indicator bacteria such as total coliform, fecal coliform, and *Pseudomonas aeruginosa*, as well as 16S rRNA sequencing, to investigate the impact of environmental conditions and spatial variations on the diversity of bacterial communities in the coastal waters and sediments from selected sites in Qatar. The concentration levels of indicator bacteria were affected by increasing temperatures and pH, and by decreasing salinity of seawater samples. Diversity indices and the molecular phylogeny demonstrated that *Proteobacteria*, *Bacteroidetes*, and *Cyanobacteria* were the dominant phyla in all locations. The most abundant operational taxonomic units (OTUs) at the family level were from *Flavobacteriaceae* (27.07%, 4.31%) and *Rhodobacteraceae* (22.51%, 9.86%) in seawater and sediment, respectively. *Alphaproteobacteria* (33.87%, 16.82%), *Flavobacteria* (30.68%, 5.84%), and *Gammaproteobacteria* (20.35%, 12.45%) were abundant at the species level in both seawater and sediment, while *Clostridia* (13.72%) was abundant in sediment only. The results suggest that sediment can act as a reservoir for indicator bacteria, with higher diversity and lower abundance compared to seawater.

## 1. Introduction

The Gulf region, including Qatar, depends primarily on seawater desalination to meet the growing water demands. Desalination using reverse osmosis (RO) membrane technology is increasingly dominating worldwide, due to its lower energy consumption compared to multistage flash distillation and multiple-effect distillation [1]. The main challenge with RO is biological fouling (biofouling), mainly caused by bacterial growth. Biofilm’s resistance to disinfection methods during the RO pretreatment process is usually affected by site-specific bacterial behavior, which is based on diversity and interspecies relationships with the environmental conditions [2]. Therefore, it is important to determine the microbial ecology of water sources [3,4]. Most recent studies have focused on investigating the microbial community structure on membrane surfaces and understanding their role in biofouling [5,6], in order to optimize treatment efficiency and plant performance [7]. Few studies have discussed the importance of tailoring the treatment processes based on the type of planktonic microbiome in the surface and groundwater [8,9]. Moreover, anthropogenic activities including desalination can have a significant impact on marine biodiversity, including marine microbial community structure. Therefore, it is important to understand the microbial community structures and the patterns of distribution in different marine environments [10]. The arid conditions of the Arabian Gulf, represented by extreme salinity and temperature, have pronounced effects on physiological aspects of the microbial community, as well as their diversity, abundance, and local distribution [11]. Changes in the seawater quality of the Arabian Gulf—including temperature, dissolved oxygen, acidity, and salt concentration—may severely affect multiple marine organisms, including bacterial communities [12,13,14]. Moreover, despite high standards of sewage treatment (i.e., secondary or tertiary) [15], large quantities of domestic effluents are discharged to coastal and marine environments in the Arabian Gulf. These effluents are characterized by high amounts of suspended solids and high loads of nutrients such as ammonia, nitrates, and phosphates [16]. To date, the literature is lacking comprehensive investigation into Qatari marine biodiversity.

Here, we aim to investigate the bacterial biodiversity and community composition in selected coastal regions of Qatar. Bacterial community composition (presence–absence and relative abundance) in the seawater and near-seafloor sediment are studied using high-throughput sequencing. Physicochemical parameters and baseline data regarding indicator organisms in the seawater and sediment along the Qatar coast are also investigated.

## 2. Materials and Methods

### 2.1. Site Descriptions and Sample Collection Protocol

Two sampling campaigns for seawater and sediment from five coastal locations of Qatar were conducted in July and September 2019. All five sites selected are near the desalination plants along the Qatar coasts. The geographical areas are depicted in Table 1.

A total of 20 samples, 10 each for seawater and sediment, were taken from five locations. Water samples were taken at 30 cm depth (to avoid the direct effect of ultraviolet radiation from the sun on the water surface layer) in sterile 1 L Pyrex bottles. Sediment samples were collected using sterile 50 mL Falcon conical screw-cap tubes, and then directly transported to the laboratory in an icebox within 2 h of collection and stored at −20 °C until further analysis.

### 2.2. Sample Preprocessing and Physicochemical Analysis

The sediment samples were pretreated as described previously [17]. Briefly, 1 g of sediment was added into 9 mL of sterile distilled water and mixed thoroughly using vertex mixing. The mixture was centrifuged at 8000 rpm for l–2 min and then was left to stand for 5–10 min to allow large particles to settle. The supernatant was subsequently used for further processing. The treated sediment and as-received seawater samples were used for genomic DNA extraction.

Physicochemical parameters of the collected water samples, including temperature, pH, salinity, dissolved oxygen (DO), and conductivity, were analyzed. The pH and temperature of seawater samples were measured on-site using pH Test Strips (VWR Chemicals BDH^®^, Batavia, IL, USA) and the Digi-Sense Thermocouple Thermometer with Calibration (Traceable^®^ Kangaroo, Batavia, IL, USA), respectively. Conductivity and DO were measured using Thermo Scientific™ Orion™ Versa Star Pro™ (Thermo Scientific^®^, Beverly, MA, USA) meters.

### 2.3. Bacteriological Analysis

Analyses of indicator microbes, including total coliform, fecal coliform, *Escherichia coli*, *Enterococci*, *Pseudomonas aeruginosa*, and bacterial Heterotrophic Plate Count (HPC), were performed according to the standard methods [18]. Colilert-18 (IDEXX, Maine, ME, USA) was employed to determine the most probable number (MPN) per 100 mL of total coliforms, fecal coliforms, and *E. coli*. Enterolert (IDEXX, Maine, ME, USA) was used to detect MPN per 100 mL of *Enterococci.* The five-tube multiple-dilution technique was used to determine *P. aeruginosa* as a colony-forming unit (CFU) per mL. HPC bacteria were studied using the spread plate technique as CFU/mL.

### 2.4. DNA Extraction, PCR Amplification, and 16S rRNA Amplicon Sequencing

Total genomic DNA was extracted from seawater and sediment samples using the PureLink™ Genomic DNA Mini kit (Invitrogen, Carlsbad, CA, USA) according to the manufacturer’s instructions. The DNA samples were then quantified using the Qubit dsDNA High Sensitivity Assay Kit (Invitrogen, USA). The Amplification of the V1-V3 region of bacterial 16S rRNA genes was carried out with the NEXTFLEX^®^ 16S V1-V3 Amplicon-Seq Kit (PerkinElmer, Austin, TX, USA) [19], according to the manufacturer’s instructions. Briefly, 5 ng of genomic DNA was used for the initial PCR amplification using customized PCR primers that target the V1-V3 domains. The products were purified, and the quality was analyzed. Next, the second PCR amplification was performed to integrate the Illumina flow cell binding domains and the unique 12 base pair sample indices using 18 cycles. These products were cleaned, and their quality and quantity were analyzed. The 31 pooled 16S V1-V3 libraries were sequenced using the Illumina MiSeq V3 reagent kit (2 × 300) with ~5% PhiX control. QIIME (Boulder, CO, USA) software QIIME 2 was used for taxonomic richness estimates, statistical analysis, inter-sample comparability, total operational taxonomic units (OTUs), Chao-1, ACE, and phylogenetic diversity (PD) [20].

## 3. Results and Discussion

This study was conducted in 2019 by collecting samples of seawater and sediment from five different coastal locations in Qatar. The objective of the study was to analyze the microbial structure and diversity of the samples, as well as to investigate the relationships between bacterial communities and both seawater and sediment. The microbial analysis was performed by targeting the V1-V3 regions of the 16S rRNA gene. Additionally, the study examined the correlations between bacterial indicators—such as total coliform, fecal coliform, *P. aeruginosa*, and HPC—and various physicochemical parameters.

### 3.1. Environmental Factors and Their Relationships with Marine Bacterial Communities

Understanding the impact of environmental changes on bacterial community biodiversity and composition is important. Physicochemical variables, including temperature, salinity, pH, and nutrients—along with geographical location, seasonality, light availability, water depth, and tide—affect gene abundance and diversity in bacteria [21,22] and can play a significant role in the formation and composition of biofilm in marine environments [23,24].

The physicochemical analysis of seawater samples from the five coastal sites is presented in Table 2. In brief, the temperature of the seawater in July ranged between 31 ± 0.18 °C and 37 ± 0.19 °C across all selected sites, whereas in September it ranged from 21.4 ± 0.18 °C to 24 ± 0.19 °C. The pH was slightly alkaline, with values ranging from 8.24 ± 0.1 to 8.60 ± 0.1 during July and between 8.26 ± 0.1 and 8.60 ± 0.1 in September, showing that there was not much difference in alkalinity. Samples from Wakra had the highest alkalinity during both times, and the lowest pH was observed at Dukhan. The lowest conductivity of the seawater samples was observed in samples taken from The Pearl during July (the value was 60.0 ± 0.36 ms/Cm). Meanwhile, the highest conductivity was noted in Dukhan during September (80.4 ± 0.36 ms/Cm). The physicochemical characteristics from sampling sites indicate that the seawater at the selected locations in coastal Qatar is warm and alkaline.

### 3.2. Indicator Bacteria Concentrations in Seawater

**Figure 1:**
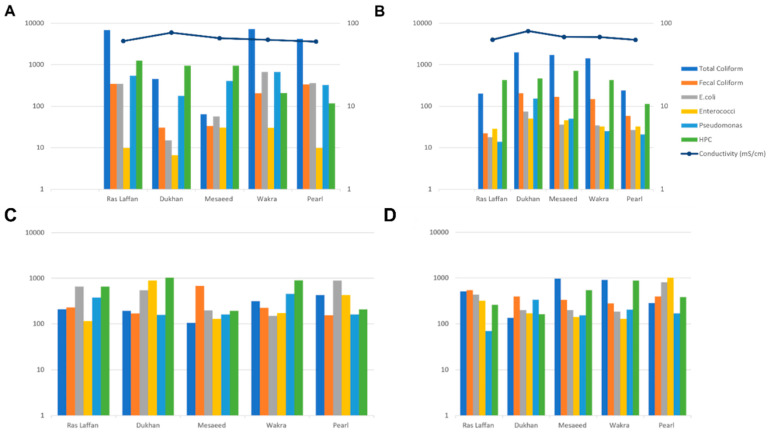
The incidence of indicator bacteria in seawater samples between all locations ((**A**)—during July, (**B**)—during September), and the incidence of indicator bacteria in sediment samples between all locations ((**C**)—during July, (**D**)—during September).

The concentration levels of indicator microorganisms (total coliform, fecal coliform, *E. coli*, *Enterococci*, *P. aeruginosa*, and HPC) during July and September are presented in Figure 1A and Figure 1B, respectively. In July, the presence of indicator bacteria varied across different locations, with Wakra experiencing the highest incidence. Specifically, Wakra had elevated levels of total coliform and *E. coli*, while *Enterococci* was found in both Wakra and Mesaeed. In July, the spatial distribution of indicator bacteria exhibited significant spatial heterogeneity among the various monitored locations. Notably, Wakra emerged as the site with the most prominent prevalence of these indicator bacteria, characterized by substantially elevated concentrations of both total coliform and *E. coli*. In contrast, the presence of Enterococci demonstrated uniform distribution, being equally prevalent in both Wakra and Mesaeed. Moreover, the incidence of Pseudomonas aeruginosa was consistently detected in equal measures in Wakra and Ras Laffan, indicating a shared prevalence of this particular microorganism. On the contrary, Ras Laffan and Pearl exhibited comparable elevated levels of fecal coliform, suggesting a similar degree of contamination in these two locations. Furthermore, Ras Laffan also presented notably heightened levels of HPC, signifying an increased microbial load in its waters.

Conversely, Dukhan had the lowest incidence of indicator bacteria in seawater, with lower levels of fecal coliform, *E. coli*, *Enterococci*, and *P. aeruginosa*. Mesaeed showed low levels of total coliform, and Pearl had lower levels of HPC. During September, the highest incidence of all indicators under study was observed at Dukhan, except HPC, the highest levels of which were recorded at Mesaeed. These findings indicate a positive association between the occurrence of indicator bacteria and the conductivity of seawater samples. On the other hand, there was a negative association between conductivity and HPC bacteria. Consequently, our data revealed that with increasing conductivity, indicator microbes in seawater samples increased. In addition, we observed a positive association between temperature, pH, and indicator microorganisms, but only within specific ranges and not in all cases. However, another research group detected non-statistically significant relationships between indicators (total coliforms, fecal coliforms, and *Salmonellae*) and temperature, pH, turbidity, and suspended solids in seawater [25]. Temperature significantly impacted the survival of indicator bacteria (coliforms and fecal coliform bacteria, and fecal *Streptococci*) [26]. In these extreme environments, the interaction between factors such as temperature, salinity, and pH results in the colonization of microbes that have adapted to these conditions [27].

### 3.3. Indicator Bacteria Concentrations in Sediment

Figure 1C,D depict the concentration of indicator bacteria (total coliform, fecal coliform, *E. coli*, *Enterococci*, *P. aeruginosa*, and HPC) in sediment samples from the Qatar coastal area during July (Figure 1C) and September (Figure 1D). In July, Pearl had the highest incidence of total coliform in sediment samples among all locations, while Mesaeed displayed the highest incidence during September. Mesaeed also exhibited the highest incidence of fecal coliform in July, whereas Ras Laffan had the highest incidence in September. For *E. coli*, Pearl recorded the highest incidence during both time periods. In July, Dukhan experienced the highest levels of Enterococci, whereas Pearl had the highest incidence in September. *P. aeruginosa* showed high levels at both Ras Laffan and Wakra in July, but in September, Dukhan exhibited elevated levels. HPC was elevated in Dukhan during July and in Wakra during September.

At all the studied sites, not all indicator bacteria are more abundant in sediment than in seawater. Earlier studies have reported that there is no correlation between water column bacteria and sediment [28,29]. As indicator microbes are used to monitor fecal contamination [30], an increase in the number of indicator bacteria in seawater and sediment is associated with a greater risk of pathogenic microorganism-induced illness in humans [31]. Within aquatic systems, the indicator microorganisms can be highly related to sediment fraction [32]. In general, indicator bacteria can survive much longer in sediment than in the water column in both freshwater and seawater environments [17].

### 3.4. Diversity and Taxonomic Composition of Marine Bacteria at Qatar Coastal Locations

**Figure 2:**
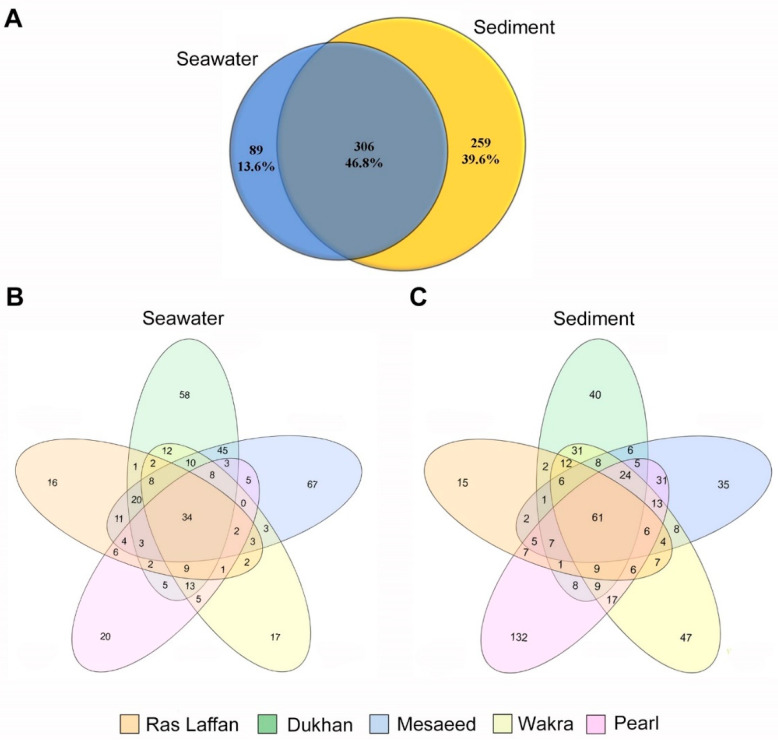
Venn diagrams of overlapping OTUs for marine bacterial communities, denoting the number of OTUs corresponding to each percentage of unique and shared species between seawater and sediment samples. The indicated numbers in the Venn diagrams are extracted from the rarefied OTU abundance tables (depending on spp. level). (**A**) indicates seawater and sediment samples, (**B**) seawater samples, and (**C**) sediment samples from different coastal locations in Qatar.

In order to determine the distribution and composition of microbiota in the seawater and sediment samples, next-generation sequencing (NGS) was performed on the collected samples using the 16S rRNA amplicons from bacterial DNA. The readings from seawater and sediment were taxonomically clustered into 97% of sequences identified as operational taxonomic units (OTUs). Following the sequencing analysis, a total of 654 OTUs were identified. Figure 2A displays the OTU counts—obtained using MiSeq sequencing—for the seawater and sediment samples, which were 395 and 565, respectively. Among these, 306 OTUs were found to be shared between the seawater and sediment samples, indicating a degree of overlap in species composition. Furthermore, there were 89 unique OTUs specific to the seawater samples, and 259 unique OTUs specific to the sediment samples, indicating distinct species diversity at the species (spp.) level within each sample type. Figure 2B,C depict data of shared and unique OTUs from the five sites. The number of OTUs in the seawater sample taken from Ras Laffan was 124; 233 for the sample from Dukhan; 226 for Mesaeed; 129 for Wakra; and 120 for the Pearl (Figure 2B). On the other hand, the number of OTUs in sediment samples according to site location were 151 at Ras Laffan; 230 at Dukhan; 222 at Mesaeed; 268 at Wakra; and 341 at The Pearl (Figure 2C). These data show that the diversity and richness index of the microbiota associated with the different locations was higher in the sediment samples. Based on the nonparametric Chao1 and Shannon indices, the known richness of the entire marine bacterial community showed higher bacterial diversity in sediment than in seawater samples. NGS analysis using 16r RNA is one way to identify common organisms in biofilms, and OTU classifications can be achieved with this identification method when strains bear less than 97–98% similarity [33]. Below 97% similarity, organisms must be differentiated via alternative techniques, such as DNA–DNA hybridization [2,34,35].

### 3.5. Microbial Composition and Distribution in Seawater and Sediment Samples

We studied the relative abundance and diversity of the marine bacterial communities in the coastal marine samples using 16S rRNA sequencing at phylum, family, and genus levels. The resulting readings were assigned to bacterial phyla in each sample, based on alignment with the SILVA database; 654 OTUs were identified at different taxonomic levels and distributed across 1 kingdom, 47 phyla, 132 classes, 238 orders, 379 families, and 594 genera.

**Figure 3:**
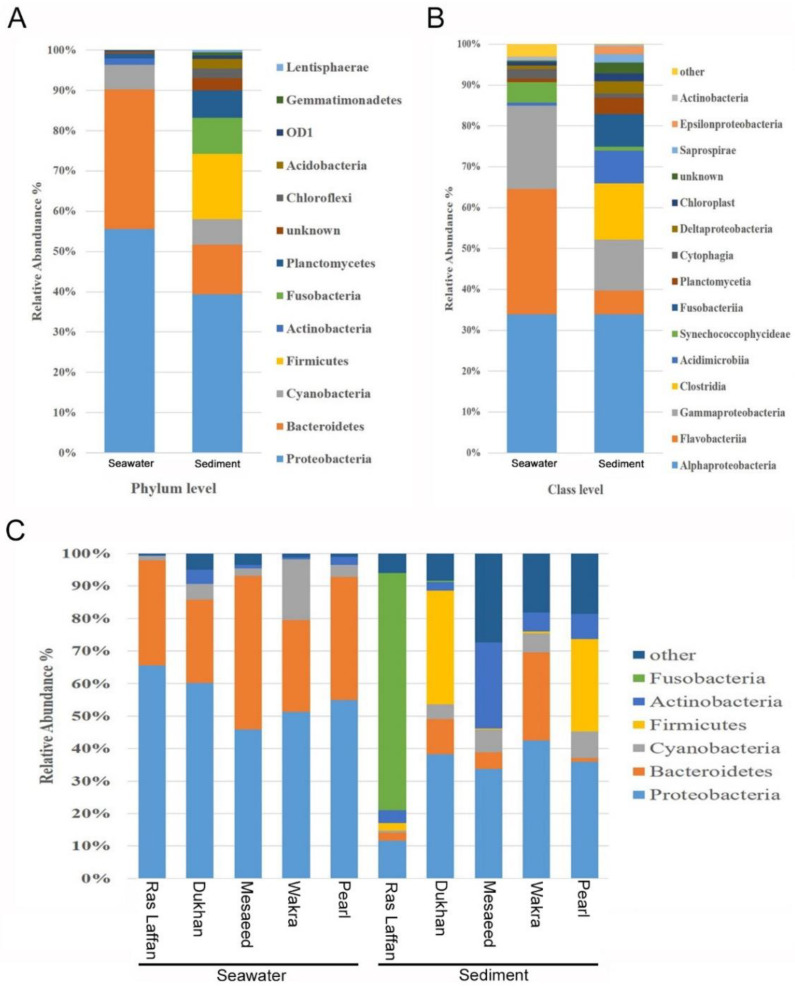
Marine bacterial composition profiles in seawater and sediment. Relative phylogenetic abundance was based on the frequencies of 16 SrRNA gene sequences affiliated with major bacterial phyla and class. The bacterial compositions of both seawater and sediment are shown at (**A**) phylum level and (**B**) class level. Data indicates the difference in richness between seawater and sediment samples. (**C**) The bacterial compositions of both seawater and sediment, from five different locations, at the phylum level.

At the phylum level, all five locations showed a similar microbial community structure, with *Proteobacteria, Bacteroidetes, Cyanobacteria, Firmicutes, Actinobacteria, Fusobacteria, Planctomycetes, Chloroflexi, Acidobacteria, Gemmatimonadetes, Lentisphaerae, Verrucomicrobia, Tenericutes, Spirochaetes, Nitrospirae*, and *Thermotogae* making up the top sixteen ubiquitous phyla. Among these identified phyla, the relative abundance of *Proteobacteria* and *Bacteroidetes* in the seawater samples were 55.41% and 34.55%, respectively. Whereas, in the sediment samples, the highest relative abundance was of *Proteobacteria* and *Firmicutes*, with an average composition of 34.94% and 14.39%, respectively, as depicted in (Figure 3A). The taxonomic classification revealed that phylum *Proteobacteria* was the most dominant in the marine bacterial communities in all seawater and sediment samples, followed by *Bacteroidetes* and *Cyanobacteria*. The phylum *Firmicutes* and *Fusobacteria* were detected only in sediment samples, as shown in Figure 3A. Furthermore, class-level data analysis showed that the marine bacterial communities were more abundant in sediment than in seawater, as displayed in Figure 3B. Seawater microbiota was dominated primarily by the *Alpha proteobacteria* class (33.87%), followed by *Flavobacteria* (30.68%), and *Gamma proteobacteria* (20.35%), with most of the readings belonging to the phyla *Proteobacteria* and *Bacteroidetes*. In the sediment, microbiota comprised a relative abundance of *Alpha proteobacteria* class (33.87%), followed by *Clostridia* (13.72%), *Gamma proteobacteria* (12.46%), *Acidimicrobia* (8.05%), *Fusobacteria* (8%), and *Flavobacteria* (5.84%). *Clostridia* and *Fusobacteria* were observed only in sediment samples, whereas *Vibrionaceae* and *Pseudoalteromonadaceae* classes were identified in both seawater and sediment samples. However, for both classes, their relative abundance in seawater samples was higher than in sediment samples. For instance, Vibrionaceae showed a relative abundance of 13.22% in seawater, a value which was higher than that found in the sediment samples (3.24%).

Abundance-weighted community compositions of sediment samples revealed a higher diversity and taxonomic richness than seawater samples at all five locations. The number of OTUs in the sediment samples was three times higher than in seawater samples. The results obtained in this study are in line with earlier reports, where it is stated that microbial diversity increases along the water column, with the lowest rates at the water surface and the highest in sediments [36,37]. Seafloor sediments consist of microbes from particles sinking from overlying seawaters, leading to a certain resemblance between seawater and sediment microbial communities [38]. Sea sediments could have higher microbial diversity due to four reasons: (1) the sediment provides a place for microbial attachment; (2) it serves as a reservoir for favorable organic substances and nutrients for microbes; (3) it protects from environmental stresses such as UV radiation [39]; and (4) it enriches protozoan grazing and the formation of extracellular polymeric substances (EPS) [40,41].

Clostridia are found in sediments because they thrive in environments with little or no oxygen, and sediments typically provide these oxygen-poor conditions. Additionally, they can break down organic materials in the sediments, and the nutrients in sediments serve as a food source for them [42]. The higher diversity of bacteria in sediments can be connected to urban pollution, particularly through the accumulation of settling particulate matter. Urban areas release various pollutants into the environment, which, upon settling in sediments, create conditions that support a wide range of bacterial species. These pollutants can serve as a nutrient source and a surface for bacterial growth. Understanding this link between urban pollution and bacterial diversity in sediments is essential for assessing the impact of human activities on aquatic ecosystems.

As shown in Figure 3C, the highest relative abundance of the phylum *Proteobacteria* was noticed in the seawater samples from Ras Laffan (65.56%), and this phylum accounted for a large proportion of the bacteria at all sites. The relative abundance in Dukhan was (60.18%), followed by Pearl (54.90%), Wakra (51.28%), and Mesaeed (45.92%); but the highest relative abundance in the sediment samples was noticed at Wakra (42.54%), followed by Dukhan (38.324%), Pearl (35.95%), Mesaeed (33.69%), and Ras Laffan (11.69%). The *Bacteroidetes* phylum was highly represented in the seawater samples from Mesaeed (47.13%), Pearl (37.97%), Ras Laffan (32.32%), Wakra (28.31%), and Dukhan (25.57%). It was also highly represented in the sediment samples from Wakra (27.07%), Dukhan (10.74%), and Mesaeed (5.12%). In addition, *Fusobacteria* was present with high relative abundance in the Ras Laffan sediment sample (73%), while in the sediment samples from other sites it was noticed only in small proportions. The *Fusobacteria* phylum predominated in the sediment sample from Ras Laffan. Also, *Firmicutes* were detected at a higher relative abundance at Dukhan (34.95%), followed by lower concentrations at Pearl (28.39%) and Ras Laffan (2.34%). The phylum *Cyanobacteria* was highly represented in the seawater sample from Wakra (18.68%), followed by those from Dukhan (4.92%), Pearl (3.61%), Mesaeed (2.44%), and Ras Laffan (1.34%). The phylum *Cyanobacteria* was highly recognized in the sediment samples from Pearl (8.27%), Mesaeed (7.13%), Wakra (5.76%), Dukhan (4.53%), and Ras Laffan (0.65%). In addition, the phylum *Actinobacteria* showed higher abundance in the sediment samples rather than the seawater samples across all locations. The 16S rRNA gene metabarcoding analysis shows that *Proteobacteria* is a dominant bacteria in the biofouling of most reverse osmosis membranes, as well as ß-*Proteobacteria* in severely fouled membranes [43]. During a study to identify the bacteria responsible for membrane biofouling in seawater reverse osmosis, samples were collected near the Ras Laffan, Dukhan, Mesaeed, Wakra, and Pearl seawater RO plants. The results showed that bacterial populations in the source seawater and biofilms were significantly different depending on location and season [5]. The dominant alpha *Proteobacteria* genera were in the *Rhodobacterales* clade, which is known to be a key biofilm-forming species in coastal and offshore marine waters throughout the temperate and polar areas of the globe [44,45]. There is, however, limited information on the detection or dominance of this bacterial clade within biofilms developed in subtropical offshore waters. The *Rhodobacterales* contain diverse physiological characteristics, including quorum sensing, hydrocarbon degradation, secondary metabolite production, gene transfer agents, and carbon monoxide oxidation [45,46]. In marine environments, *Gamma proteobacteria* and *Bacteroidetes* are the pioneers of biofilm formation [40]. Previous studies have shown that subsurface waters have a higher abundance of free-living cyanobacteria than deep waters [39,41]. In addition to *Cytophaga* and *Flavobacteria*, the other two major classes of *Bacteroidetes* are important congregates of organic detritus and marine aggregates [47].

As we observed a significant difference between the microbial populations at the five sites along the coast of Qatar, the dominant phylum (*Proteobacteria*) exhibited a high relative abundance in both the seawater sample from Ras Laffan and the sediment sample from Wakra. Dukhan ranked second among all locations for the abundance of *Proteobacteria*. A slight change in location within a specific area can significantly alter the composition and dominance of biofilm-forming species, thereby impacting the consequences of urban pollution. Furthermore, all samples contained a large number of unknown species—especially the sediment sample from the Mesaeed location, followed by the sediment samples from Wakra and Pearl—which provides new information relevant to the discovery of potential new microbial species.

**Figure 4:**
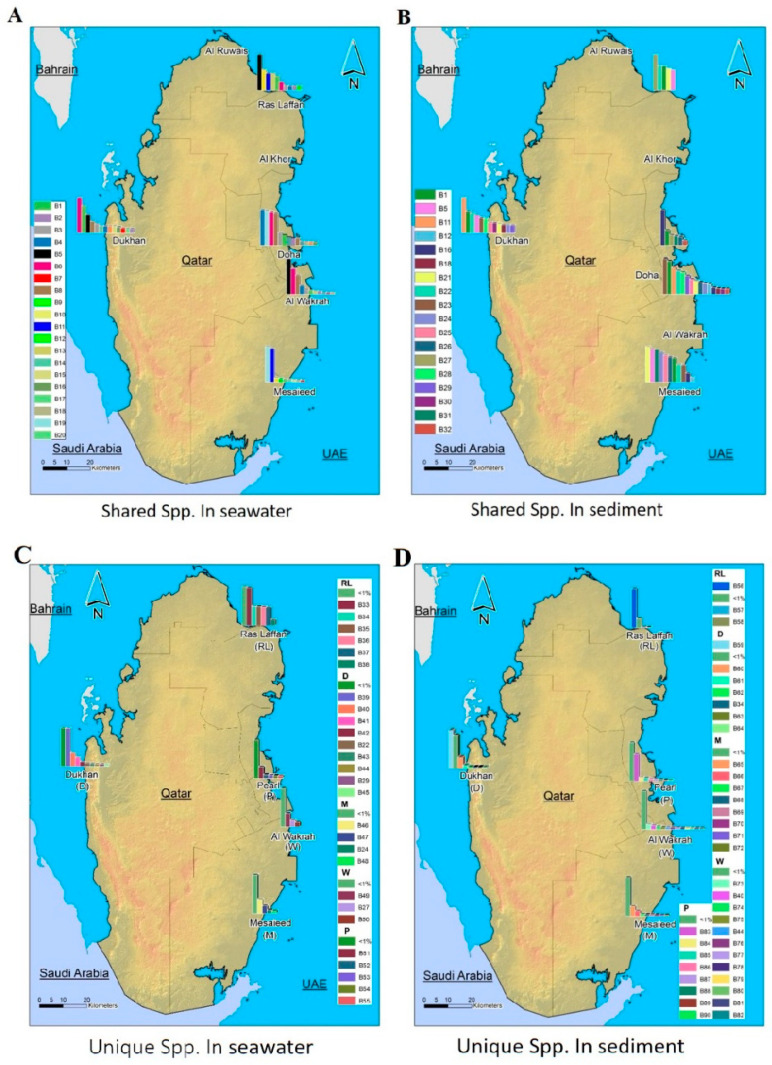
Qatar maps showing the spatial distribution patterns of representative phylogenetic groups according to the relative abundance of bacterial taxa, which are determined at taxonomic levels by species (spp.). (**A**): the relative abundance of coastal marine bacterial spp. shared across seawater samples from all locations. (**B**): the relative abundance of coastal marine bacterial spp. shared across sediment samples from all locations. (**C**): the relative abundance of coastal marine bacterial spp. unique to seawater samples from different locations. (**D**): The relative abundance of coastal marine bacterial spp. unique to sediment samples from different locations. (Names and coding related to them are displayed in Supplementary Table S1).

Figure 4 depicts the spatial distribution pattern of representative phylogenetic groups according to the relative abundance of bacterial taxa, which is determined at species level. The full classification of bacteria from kingdom to species level is presented in Supplementary Table S1.

The spatial distribution and composition of the marine microbes depend on the complex ecological and evolutionary processes in the ecosystem. MiSeq data analysis revealed distinct differences in bacterial community structures according to geographical location and the nature of the samples.

Some studies have shown that microbes that are rare in one location may be dominant in others, or dominant at the same location with different environmental conditions [10]. In this study, we discovered that the abundance-weighted compositions of the soil and water bacterial communities exhibited minimal differences. We observed that the primary phylum (*Proteobacteria, Bacteroidetes*, and *Cyanobacteria*) present in both samples was consistent. We have examined the extent of taxonomic overlap between the seawater and sediment communities of the pooled samples collected from all locations during the sample collection period (July), which is consistent with other studies in different geographic locations [48,49,50]. Furthermore, we observed differences between locations, which led to additional questions related to bacterial ecology and biodiversity. Microbial transport through the water column may explain large-scale biogeographic patterns in near-seafloor sediments as well as subseafloor sediments [51].

### 3.6. Structural Analysis of the Microbial Communities in the Samples

**Figure 5:**
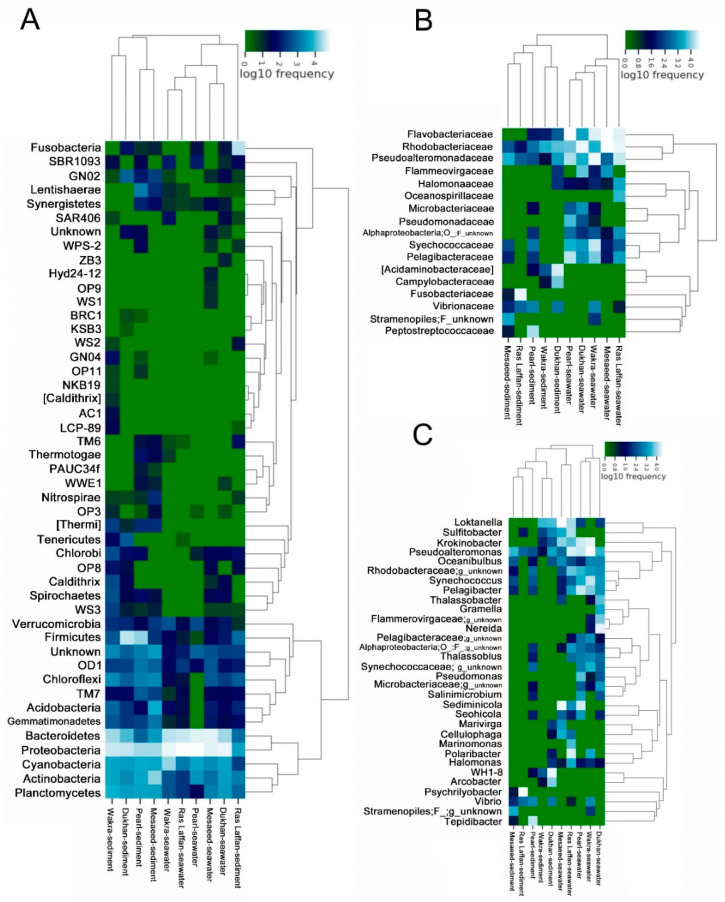
Heat map clustering visualization of seawater and sediment bacterial communities into phylum levels. (**A**) Diagram showing the relative abundance of OTUs in bacterial communities from the five coastal areas (seawater and sediment samples) according to the phylum level. The relative abundance of bacterial composition in the seawater and sediment is shown at the (**B**) Family level and (**C**) Genus level.

In order to understand the similarities between different samples and site locations, we performed a cluster analysis of these samples and constructed a sample cluster tree. The clustering results were integrated with the species’ relative abundance column chart at the phyla taxon level for each sample (Figure 5A), the relative abundance column chart at the family taxon level (Figure 5B), and the relative abundance column chart at the species taxon level (Figure 5C).

The cluster analysis performed using phylum showed that different sites had significantly different marine microbial community structures. We observed that *Proteobacteria* and *Bacteroidetes* were dominant in all samples. Moreover, the cluster analysis performed using family levels showed that *Flavobacteriaceae*, *Rhodobacteraceae*, *Pseudoalteromonadaceae*, *Pelagibacteraceae*, *Acidaminobacteraceae*, and *Synechococcaceae* were abundant in all samples from different locations. In the current study, most of the dominant *Alphaproteobacterial* genera were found within the *Rhodobacterales* class. *Rhodobacteriaceae* and *Flavobacteriaceae* are dominant in the biofilm-forming community structure, with distinct groups assembling according to the surface type or immersion method [52]. Similarly, in numerous locations of the Atlantic and Pacific Oceans, *Rhodobacteraceae* dominate the biofilm component [53,54]. Several unknown species were also identified in all samples.

### 3.7. Alpha and Beta Diversity of the Microbial Communities

**Figure 6:**
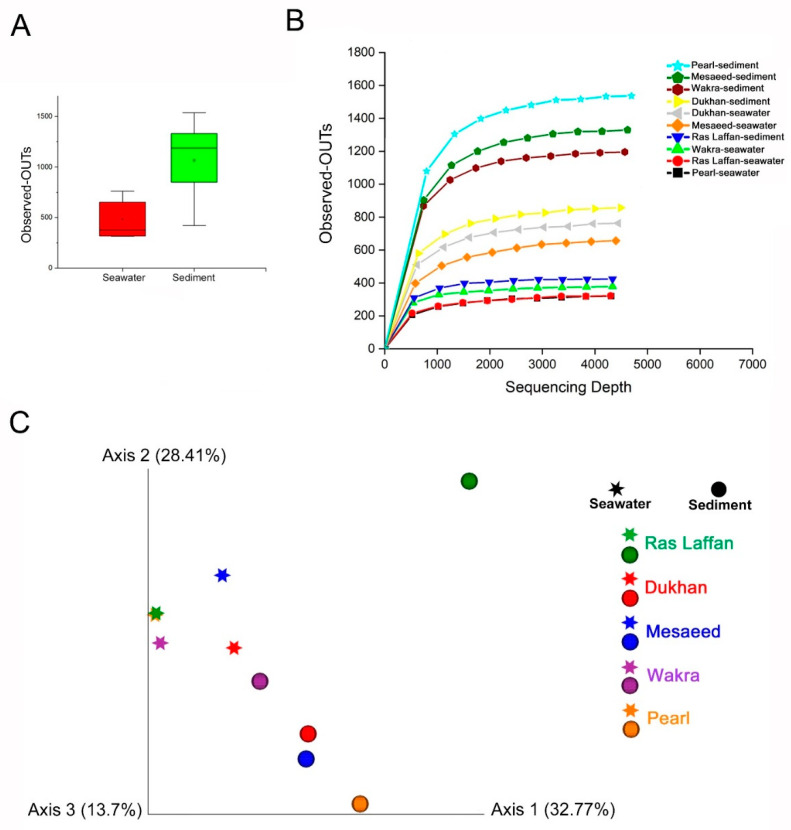
Alpha diversity shows the diversity in a single ecosystem (each sample). Where the Alpha diversity is the simplest measure, richness (the number of species or distinguishable taxa (OTUs)) is observed in each sample. (**A**) The rarefaction curves and the number of observed OTUs, where the abundance of species of bacteria in sediments is higher than in seawater. (**B**) Species richness is the number of different species in a sample. (**C**) PCoA plots (by sample type). Samples—each colored according to the sample location and shaped according to sample type—show less defined clustering in the PCoA scatter plot, calculated using the Bray–Curtis dissimilarity. The first two principal component analyses, PC1 and PC2, explained 32.77% and 28.41% of the variations, respectively.

Bacterial communities’ alpha diversity and beta diversity are used to examine variations in community structure across environmental gradients, space, and time [55]. Maintaining bacterial alpha diversity in natural and managed ecosystems is crucial for both the function and stability of ecosystem processes [56]. Figure 6 presents the bacterial taxa richness (alpha diversity) and community composition (beta diversity) used to evaluate the marine bacterial composition and diversity related to all locations. Alpha and beta diversities were calculated using different indices. Our analysis showed that the collected samples’ bacterial community structures have high alpha diversity. Figure 6A shows that the abundance of OTUs in sediment samples is higher than in seawater samples, whereas Figure 6B provides more details based on the location. Our findings indicated a high level of OTUs in the sediment sample taken from the Pearl, followed by those from Mesaeed, Wakra, and Dukhan, with the lowest level of OTUs found in the sediment taken from Ras Laffan. Furthermore, the OTUs in Dukhan seawater, followed by those in Mesaeed seawater, are higher than those found in Ras Laffan sediment. Ras Laffan seawater and Pearl seawater have the lowest level of OTUs. The rarefaction curves of all the collected samples were generated using QIIME software (Version 1.7.0) and normalized to the minimum number of sequences. Moreover, regarding the alpha diversity, neither the Shannon nor Simpson diversity indices showed significant differences among the locations. There were no significant differences between all the calculated alpha diversity indices—including the observed OTUs, Shannon’s diversity, Chao richness, and Heip’s evenness (*p*  >  0.05)—for either the seawater or the sediment samples, which indicated that the nature of the sample and the location are essential factors that could affect marine microbial diversity.

Here we compared the overall microbial diversity between groups using the weighted and unweighted UniFrac distance matrices. Principal Coordinates Analysis (PCoA) was used to visualize the beta diversity results (between-sample diversity comparison). The seawater samples were found to be far apart from the sediment samples (Figure 6C), and PCoA reflected the remarkable variability of the seawater samples, with a first axis (PC1) showing 32.77% of the variation, and a second axis (PC2) showing 28.41% of the variation within the sampling site. Furthermore, sediment from Ras Laffan showed a high level of beta diversity. The results of the study indicated that the nature of samples and their locations can affect the marine microbial community, which was confirmed by the distance between clusters of samples. Zinger et al. (2011) investigated the bacterial beta diversity of seafloor and seawater ecosystems, and they found that the pelagic and epibenthic communities differed greatly at all taxonomic levels [57].

## 4. Conclusions

This work investigated the indicator bacteria profiles plus the bacterial diversity of seawater and sediment in the coastal area of Qatar, by combining traditional culturing and detection techniques with 16S rRNA gene sequencing of the seawater and sediment samples. Our results revealed a strong impact of both the sampling location and the environment sampling type on the microbial population and density. Also, the results highlight the importance of spatial and temporal sample continuity in understanding bacterial biodiversity patterns. The occurrence of indicator bacteria provides context for changes induced by urban development. *Proteobacteria* and *Bacteroidetes* are the dominant phyla in the five locations in Qatar surveyed during this study. The bacterial groups *Proteobacteria*, *Bacteroidetes*, and *Cyanobacteria* were found in all samples of seawater and sediment. Meanwhile, the phylum *Firmicutes* was found in sediment samples only. Sediment samples collected near the Pearl desalination plant have the highest OTUs (341) and the lowest OTUs (120) of the seawater samples. A high number of unclassified sequences were also detected; further studies are required to identify those microorganisms. There was a reverse correlation between the concentration levels of indicator bacteria and the salinity of seawater. The incidence of indicator organisms was higher in sediment samples than in seawater samples. The order of indicator bacteria in seawater and sediment samples was HPC > total coliform > fecal coliform > *E. coli* > *Enterococci* > *P. aeruginosa*. In summary, our results indicate that the microbial concentration and diversity in the marine coastal areas along the Arabian Gulf in Qatar are significantly influenced by various environmental factors, and evaluating the influence of anthropogenic activities on microbial communities requires rigorous statistical analysis in order to explore the intricate connections between water environmental conditions and microbial populations. Subsequent research endeavors will encompass comprehensive data collection and advanced statistical methodologies to deepen our understanding of these complex relationships.

## Figures and Tables

**Table 1:** The geographical locations of the five seawater reverse osmosis (SWRO) plants under investigation.

Location	Geographical Location	
	Latitude	Longitude
Ras Laffan (RL)	25°57′15.7″ N	51°29′23.5″ E
Dukhan (D)	25°24′57.6″ N	50°45′30.2″ E
Mesaeed (M)	24°51′17.4″ N	51°30′43.5″ E
Wakra (W)	25°11′07.3″ N	51°37′12.7″ E
Pearl (P)	25°22′12.5″ N	51°31′44.3″ E

**Table 2:** Environmental parameters of the sampling locations.

Sampling Time	Location	Temp. (°C)	pH	Conductivity (mS/cm)
July	Ras Laffan	37 ± 0.19	8.27 ± 0.1	60.9 ±0.36
	Dukhan	31 ± 0.18	8.24 ± 0.1	76.9 ± 0.36
	Mesaeed	34 ± 0.18	8.28 ± 0.1	65.9 ± 0.36
	Wakra	37 ± 0.19	8.60 ± 0.1	63.2 ± 0.36
	Pearl	36 ± 0.19	8.24 ± 0.1	60.0 ± 0.36
September	Ras Laffan	21.4 ± 0.18	8.26 ± 0.1	63.3 ± 0.36
	Dukhan	22.1 ± 0.18	8.23 ± 0.1	80.4 ± 0.36
	Mesaeed	22.5 ± 0.18	8.40 ± 0.1	68.3 ± 0.36
	Wakra	24 ± 0.19	8.60 ± 0.1	68.2 ± 0.36
	Pearl	22 ± 0.18	8.31 ± 0.1	63.0 ± 0.36

## Data Availability

The data that support the findings of this study are available from the corresponding author upon reasonable request.

## Supplementary Materials

The following supporting information can be downloaded at: https://www.mdpi.com/article/10.3390/microorganisms11122827/s1, Table S1: Names and related coding.
