# Supplementary material for: Marine Bacterial Community Structures of Selected Coastal Seawater and Sediment Sites in Qatar
Source: Microorganisms. 2023 Nov 21;11(12):2827. doi: 10.3390/microorganisms11122827 (PMC10745943; doi:10.3390/microorganisms11122827)
Supplement: Supplementary file 1 [file microorganisms-11-02827-s001.zip › microorganisms-2690716-supplementary.pdf]

Marine Bacterial Community Structures of Selected Coastal Seawater and Sediment Sites in Qatar

Shimaa S. El-Malah [1], Kashif Rasool [1], Khadeeja Abdul Jabbar [1], Muhammad Umar Sohail [2], Husam Musa Baalousha [3] and Khaled A. Mahmoud [1,*]

[1] Qatar Environment and Energy Research Institute (QEERI), Hamad Bin Khalifa University, Qatar Foundation, Doha P.O. Box 34110, Qatar; salielmallah@hbku.edu.qa (S.S.E.-M.); krasool@hbku.edu.qa (K.R.); kabduljabbar@hbku.edu.qa (K.A.J.)
[2] Proteomics Core, Weill Cornell Medicine, Doha P.O. Box 24144, Qatar; mus4008@qatar-med.cornell.edu
[3] Department of Geosciences, College of Petroleum Engineering and Geosciences, King Fahd University of Petroleum and Minerals (KFUPM), Dhahran 31261, Saudi Arabia; husam.baalousha@kfupm.edu.sa
* Correspondence: kmahmoud@hbku.edu.qa

Supporting information

Table S1: Names and related coding:

Share Spp. at the water samples	code
k__Bacteria;p__Proteobacteria;c__Alphaproteobacteria;o__Rhodobacterales;f__Rhodobacteraceae;__;	B1
k__Bacteria;p__Proteobacteria;c__Alphaproteobacteria;o__;f__;g__;s__	B2
k__Bacteria;p__Bacteroidetes;c__Flavobacteriia;o__Flavobacteriales;__;__;__	B3
k__Bacteria;p__Proteobacteria;c__Alphaproteobacteria;o__Rickettsiales;f__Pelagibacteraceae;g__Pelagibacter;s__ubique	B4
k__Bacteria;p__Proteobacteria;c__Gammaproteobacteria;o__Vibrionales;f__Pseudoalteromonadaceae;g__Pseudoalteromonas;s__	B5
k__Bacteria;p__Bacteroidetes;c__Flavobacteriia;o__Flavobacteriales;f__Flavobacteriaceae;g__Krokinobacter;s__genikus	B6
k__Bacteria;p__Proteobacteria;c__Alphaproteobacteria;o__Rhodobacterales;f__Rhodobacteraceae;g__Oceanibulbus;s__indolifex	B7
k__Bacteria;p__Cyanobacteria;c__Synechococcophycideae;o__Synechococcales;f__Synechococcaceae;g__Synechococcus;s__	B8
k__Bacteria;p__Proteobacteria;c__Gammaproteobacteria;o__Alteromonadales;f__Alteromonadaceae;g__Marinobacter;s__	B9

k__Bacteria;p__Proteobacteria;c__Alphaproteobacteria;o__Rhodobacterales;f__Rhodobacteraceae;g__Sulfitobacter;__	B10
k__Bacteria;p__Proteobacteria;c__Alphaproteobacteria;o__Rhodobacterales;f__Rhodobacteraceae;g__Loktanella;s__	B11
k__Bacteria;p__Proteobacteria;c__Alphaproteobacteria;o__Rickettsiales;f__mitochondria;g__;s__	B12
k__Bacteria;p__Bacteroidetes;c__Flavobacteriia;o__Flavobacteriales;f__Flavobacteriaceae;g__Polaribacter;s__	B13
k__Bacteria;p__Bacteroidetes;c__Cytophagia;o__Cytophagales;f__Flammeovirgaceae;g__Marivirga;s__tractuosa	B14
k__Bacteria;p__Bacteroidetes;c__Flavobacteriia;o__Flavobacteriales;f__Flavobacteriaceae;__;__	B15
k__Bacteria;p__Proteobacteria;c__Alphaproteobacteria;__;__;__;__	B16
k__Bacteria;p__Bacteroidetes;c__Flavobacteriia;o__Flavobacteriales;f__Flavobacteriaceae;g__Salinimicrobium;s__	B17
k__Bacteria;p__Bacteroidetes;__;__;__;__;__	B18
k__Bacteria;p__Bacteroidetes;c__Flavobacteriia;o__Flavobacteriales;f__Flavobacteriaceae;g__Sediminicola;s__	B19
k__Bacteria;p__Proteobacteria;c__Alphaproteobacteria;o__Rickettsiales;f__Pelagibacteraceae;g__;s__	B20
k__Bacteria;p__Actinobacteria;c__Acidimicrobiia;o__Acidimicrobiales;f__koll13;g__;s__	B21
k__Bacteria;p__Planctomycetes;c__Planctomycetia;o__Pirellulales;f__Pirellulaceae;g__;s__	B22
k__Bacteria;p__Proteobacteria;c__Gammaproteobacteria;o__Thiotrichales;f__Piscirickettsiaceae;g__;s__	B23
k__Bacteria;p__Cyanobacteria;c__Chloroplast;o__Stramenopiles;f__;g__;s__	B24
k__Bacteria;p__Proteobacteria;c__Gammaproteobacteria;__;__;__;__	B25
k__Bacteria;__;__;__;__;__;__	B26
k__Bacteria;p__Proteobacteria;c__Gammaproteobacteria;o__Vibrionales;f__Vibrionaceae;g__Vibrio;__	B27

k__Bacteria;p__Bacteroidetes;c__[Saprospirae];o__[Saprospirales];f__Saprospiraceae;g__;s__	B28
k__Bacteria;p__Bacteroidetes;c__Flavobacteriia;o__Flavobacteriales;f__Flavobacteriaceae;g__;s__	B29
k__Bacteria;p__Cyanobacteria;c__Oscillatoriophycideae;o__Chroococcales;__;__;__	B30
k__Bacteria;p__Actinobacteria;c__Acidimicrobiia;o__Acidimicrobiales;f__;g__;s__	B31
k__Bacteria;p__Planctomycetes;c__Phycisphaerae;o__Phycisphaerales;f__;g__;s__	B32
k__Bacteria;p__Bacteroidetes;c__Flavobacteriia;o__Flavobacteriales;f__Flavobacteriaceae;g__Polaribacter;__	B33
k__Bacteria;p__Proteobacteria;c__Gammaproteobacteria;o__Oceanospirillales;f__Oceanospirillaceae;g__Marinomonas;s__	B34
k__Bacteria;p__Proteobacteria;c__Gammaproteobacteria;o__Oceanospirillales;f__Halomonadaceae;g__Halomonas;s__	B35
k__Bacteria;p__Bacteroidetes;c__Flavobacteriia;o__Flavobacteriales;f__Flavobacteriaceae;g__Maribacter;s__	B36
k__Bacteria;p__Bacteroidetes;c__Flavobacteriia;o__Flavobacteriales;f__Flavobacteriaceae;g__Maribacter;s__	B37
k__Bacteria;p__Proteobacteria;c__Gammaproteobacteria;o__Alteromonadales;f__HTCC2188;g__HTCC;s__	B38
k__Bacteria;p__Proteobacteria;c__Alphaproteobacteria;o__Rhodobacterales;f__Rhodobacteraceae;g__Nereida;s__ignava	B39
k__Bacteria;p__Bacteroidetes;c__Cytophagia;o__Cytophagales;f__Flammeovirgaceae;g__;s__	B40
k__Bacteria;p__Proteobacteria;c__Alphaproteobacteria;o__Rhodobacterales;f__Rhodobacteraceae;g__Thalassobacter;s__stenotrophicus	B41
k__Bacteria;p__Bacteroidetes;c__Flavobacteriia;o__Flavobacteriales;f__Flavobacteriaceae;g__Gramella;s__	B42
k__Bacteria;p__Actinobacteria;c__Actinobacteria;o__Actinomycetales;f__Microbacteriaceae;g__;s__	B43
k__Bacteria;p__Proteobacteria;c__Deltaproteobacteria;o__Myxococcales;f__;g__;s__	B44
k__Bacteria;p__Proteobacteria;c__Alphaproteobacteria;o__Rhodobacterales;f__Rhodobacteraceae;g__Amaricoccus;s__	B45

k__Bacteria;p__Proteobacteria;c__Alphaproteobacteria;o__Rhodobacterales;f__Rhodobacteraceae;g__Loktanella;s__vestfoldensis	B46
k__Bacteria;p__Bacteroidetes;c__Flavobacteriia;o__Flavobacteriales;f__Flavobacteriaceae;g__Cellulophaga;s__	B47
k__Bacteria;p__Bacteroidetes;c__Flavobacteriia;o__Flavobacteriales;f__Flavobacteriaceae;g__Salegentibacter;s__	B48
k__Bacteria;p__Cyanobacteria;c__Synechococcophycideae;o__Synechococcales;f__Synechococcaceae;__;__	B49
k__Bacteria;p__Proteobacteria;c__Alphaproteobacteria;o__Rhodobacterales;f__Rhodobacteraceae;g__Thalassobius;s__	B50
k__Bacteria;p__Proteobacteria;c__Gammaproteobacteria;o__Pseudomonadales;f__Pseudomonadaceae;g__Pseudomonas;__	B51
k__Bacteria;p__Proteobacteria;c__Alphaproteobacteria;o__Rhodobacterales;f__Rhodobacteraceae;g__Seohicola;s__saemankumensis	B52
k__Bacteria;p__Proteobacteria;c__Gammaproteobacteria;o__Alteromonadales;f__Idiomarinaceae;g__Pseudidiomarina;s__	B53
k__Bacteria;p__Proteobacteria;c__Alphaproteobacteria;o__Rickettsiales;f__Pelagibacteraceae;__;__	B54
k__Bacteria;p__Proteobacteria;c__Gammaproteobacteria;o__Alteromonadales;f__Idiomarinaceae;g__Pseudidiomarina;s__homiensis	B55
k__Bacteria;p__Fusobacteria;c__Fusobacteriia;o__Fusobacteriales;f__Fusobacteriaceae;g__Psychrilyobacter;s__	B56
k__Bacteria;p__Firmicutes;c__Bacilli;o__Lactobacillales;f__Aerococcaceae;g__Marinilactibacillus;s__psychrotolerans	B57
k__Bacteria;p__Proteobacteria;c__Gammaproteobacteria;o__Pseudomonadales;f__Moraxellaceae;g__Psychrobacter;s__	B58
k__Bacteria;p__Firmicutes;c__Clostridia;o__Clostridiales;f__[Acidaminobacteraceae];g__WH1-8;s__	B59
k__Bacteria;p__Proteobacteria;c__Epsilonproteobacteria;o__Campylobacterales;f__Campylobacteraceae;g__Arcobacter;s__	B60
k__Bacteria;p__Firmicutes;c__Clostridia;o__Clostridiales;f__[Acidaminobacteraceae];g__NP25;s__	B61

k__Bacteria;p__Proteobacteria;c__Alphaproteobacteria;o__Rhodobacterales;f__Rhodobacteraceae;g__Sulfitobacter;__	B62
k__Bacteria;p__Proteobacteria;c__Gammaproteobacteria;o__Alteromonadales;f__Alteromonadaceae;g__Marinobacter;s__	B63
k__Bacteria;p__Firmicutes;c__Clostridia;o__Clostridiales;__;__;__	B64
k__Bacteria;p__Acidobacteria;c__Sva0725;o__Sva0725;f__;g__;s__	B65
k__Bacteria;p__Actinobacteria;c__Acidimicrobiia;o__Acidimicrobiales;__;__;__	B66
k__Bacteria;p__Actinobacteria;c__Acidimicrobiia;o__Acidimicrobiales;f__C111;g__Ilumatobacter;s__fluminis	B67
k__Bacteria;p__Actinobacteria;c__Acidimicrobiia;o__Acidimicrobiales;f__JdFBGBact;g__;s__	B68
k__Bacteria;p__Chloroflexi;c__Anaerolineae;o__Ardenscatenales;f__Ardenscatenaceae;g__Ardenscatena;s__	B69
k__Bacteria;p__Bacteroidetes;c__[Rhodothermi];o__[Rhodothermales];f__Rhodothermaceae;g__;s__	B70
k__Bacteria;p__Proteobacteria;c__Gammaproteobacteria;o__Chromatiales;f__Ectothiorhodospiraceae;g__;s__	B71
k__Bacteria;p__Gemmatimonadetes;c__Gemm-2;o__;f__;g__;s__	B72
k__Bacteria;p__Bacteroidetes;c__Flavobacteriia;o__Flavobacteriales;__;__;__	B73
k__Bacteria;p__Proteobacteria;c__Deltaproteobacteria;o__Desulfobacterales;f__Desulfobacteraceae;g__Desulfococcus;s__	B74
k__Bacteria;p__Bacteroidetes;c__Flavobacteriia;o__Flavobacteriales;f__Flavobacteriaceae;__;__	B75
k__Bacteria;p__Proteobacteria;c__Gammaproteobacteria;o__Chromatiales;__;__;__	B76
k__Bacteria;p__Bacteroidetes;c__Flavobacteriia;o__Flavobacteriales;f__Flavobacteriaceae;g__Polaribacter;s__	B77
k__Bacteria;p__Proteobacteria;c__Gammaproteobacteria;o__Chromatiales;f__;g__;s__	B78
k__Bacteria;p__Bacteroidetes;c__Flavobacteriia;o__Flavobacteriales;f__Flavobacteriaceae;g__Robiginitalea;s__	B79
k__Bacteria;p__Proteobacteria;c__Deltaproteobacteria;o__Desulfobacterales;f__Desulfobulbaceae;g__;s__	B80

k__Bacteria;p__Chloroflexi;c__Anaerolineae;o__GCA004;f__;g__;s__	B81
k__Bacteria;p__Bacteroidetes;c__Flavobacteriia;o__Flavobacteriales;f__Flavobacteriaceae;g__Coccinimonas;s__marina	B82
k__Bacteria;p__Firmicutes;c__Clostridia;o__Clostridiales;f__Peptostreptococcaceae;g__Tepidibacter;s__	B83
k__Bacteria;p__Cyanobacteria;c__Synechococcophycideae;o__Synechococcales;f__Synechococcaceae;g__Synechococcus;s__	B84
k__Bacteria;p__Planctomycetes;c__Phycisphaerae;o__Phycisphaerales;f__Phycisphaeraceae;g__;s__	B85
k__Bacteria;p__Lentisphaerae;c__[Lentisphaeria];o__Lentisphaerales;f__Lentisphaeraceae;g__;s__	B86
k__Bacteria;p__Firmicutes;c__Bacilli;o__Bacillales;f__[Exiguobacteraceae];g__Exiguobacterium;s__	B87
k__Bacteria;p__Chloroflexi;c__Anaerolineae;o__SBR1031;f__A4b;g__;s__	B88
k__Bacteria;p__Cyanobacteria;c__Synechococcophycideae;o__Pseudanabaenales;f__Pseudanabaenaceae;g__Halomicronema;s__	B89
k__Bacteria;p__Proteobacteria;c__Alphaproteobacteria;o__Rhizobiales;__;__;__	B90
